# Deregulated Expression of Mitochondrial Proteins Mfn2 and Bcnl3L in Placentae from Sheep Somatic Cell Nuclear Transfer (SCNT) Conceptuses

**DOI:** 10.1371/journal.pone.0169579

**Published:** 2017-01-11

**Authors:** Marta Czernik, Paola Toschi, Federica Zacchini, Domenico Iuso, Grażyna Ewa Ptak

**Affiliations:** 1 Faculty of Veterinary Medicine, Experimental Embryology, University of Teramo, Teramo, Italy; 2 Institute of Genetics and Animal Breeding, Polish Academy of Sciences, Jastrzebiec, Poland; 3 National Research Institute of Animal Production, Balice n/Krakow, Poland; Peking University Third Hospital, CHINA

## Abstract

In various animal species, the main cause of pregnancy loss in conceptuses obtained by somatic cell nuclear transfer (SCNT) are placental abnormalities. Most abnormalities described in SCNT pregnancies (such as placentomegaly, reduced vascularisation, hypoplasia of trophoblastic epithelium) suggest that placental cell degeneration may be triggered by mitochondrial failure. We hypothesized that placental abnormalities of clones obtained by SCNT are related to mitochondrial dysfunction. To test this, early SCNT and control (CTR, from pregnancies obtained by *in vitro* fertilization) placentae were collected from pregnant ewes (at day 20 and 22 of gestation) and subjected to morphological, mRNA and protein analysis. Here, we demonstrated swollen and fragmented mitochondria and low expression of mitofusin 2 (Mfn2), the protein which plays a crucial role in mitochondrial functionality, in SCNT early placentae. Furthermore, reduced expression of the Bcnl3L/Nix protein, which plays a crucial role in selective elimination of damaged mitochondria, was observed and reflected by the accumulation of numerous damaged mitochondria in SCNT placental cells. Likely, this accumulation of damaged organelles led to uncontrolled apoptosis in SCNT placentae, as demonstrated by the high number of apoptotic bodies, fragmented cytoplasm, condensed chromatin, lack of integrity of the nuclear membrane and the perturbed mRNA expression of apoptotic genes (*BCL2* and *BAX*). In conclusion, our data indicate that deregulated expression of Mfn2 and Bcnl3L is responsible for placental abnormalities in SCNT conceptuses. Our results suggest that some nuclear genes, that are involved in the regulation of mitochondrial function, do not work well and consequently this influence the function of mitochondria.

## Introduction

Somatic Cell Nuclear Transfer (SCNT) allows the asexual reproduction of an individual by transplanting a somatic cell into an enucleated oocyte. SCNT has tremendous potential for therapeutic cloning/regenerative medicine [1], the rescue of endangered species [2], and production of transgenic animals with high efficiency [3]. Yet, cloning efficiency is currently very low, and even though there are differences between species, only 2–5% of offspring are commonly delivered [4]. Moreover, SCNT is associated with high rates of fetal and perinatal losses and abnormal offspring [5]. Our and others’ works [6–9] suggest that post-implantation mortality in cloned animals is mainly caused by placental abnormalities [7,9]. The most common placental abnormalities described in clones are placentomegaly [10], reduced vascularisation, hypoplasia of trophoblastic epithelium [7], lower numbers of binuclear cells [9,11], and altered basement membrane [12]. Thickening of the trophoblast basement membrane has been, in most cases, interpreted as a consequence of hyperplasia of the cytotrophoblast due to hypoxia. It has been shown that an early feature of hypoxia in cells is a failure of mitochondrial protein homeostasis [13]. Because mitochondria are the main respiratory organelles of the cell, produce energy, and regulate cellular metabolism, they have been the focus of much research into hypoxic injury [14,15]. Investigations into mitochondria in SCNT are limited to the mtDNA hetero/homoplasmy in the tissue of cloned offspring; no data are available for the role of mitochondria dysfunction in the developmental failure of clones.

From the mitochondrial genome, only 13 proteins are encoded in the mitochondria, while the majority (1500–2000) are encoded by the nucleus [16]. Thus, the expression of gene products is controlled, in large part, by signals provided by the nucleus and mitochondrial activity is strictly regulated by nuclear signals [17].

Mature (MII) oocytes, as well as early cleavage stage embryos, depend on the function of the mitochondrial pool present at ovulation [18], while mitochondrial replication occurs only at the blastocyst stage, once the energy demands of the embryo become higher. Consequently, any adverse influence on mitochondrial function will negatively impact the development of the pre- and post-implantation embryo. Mitochondria exhibit an interesting quality maintenance function: they have numerous periods of fusion and fission. It is known that Mfn2 plays a central role in the fusion process, but it also plays a role in key cellular functions such as oxidative metabolism, cell cycle, cell death, bringing in mitochondria to the endoplasmatic reticulum (ER), calcium (Ca^2^) homeostasis, and mitochondrial axonal transport [19]. Chan et al. found that mice deficient in Mfn2 died in mid-gestation mainly due to placental abnormalities, particularly a severe disruption of the trophoblast binuclear cell layer [20]. Besides that, it has been shown that deficient expression of Mfn2 in human trophoblastic cells might be responsible for “unexplained” early miscarriage [21]. Loss-of-function models of Mfn2 in embryonic mouse fibroblasts display distinct types of fragmented, damaged, and immature mitochondria, attributable to a marked reduction in mitochondrial function [22].

Our aim was to examine whether inadequate mitochondrial function caused placental abnormalities in SCNT placentae.

## Materials and Methods

All chemicals, unless otherwise indicated, were obtained from Sigma Chemicals Co. (St. Louis, MO, USA).

### Ethics statement

All animal experiments were performed in accordance with DPR 27/1/1992 (Animal Protection Regulations of Italy) in concordance with European Community regulation 86/609 and were approved by CEISA (Inter-Institutional Ethics Committee for Animal Experimentation) Prot. 79/2013/CEISA Prog. 58. The permit n°: CEISA VI, Classe 8.1, Prot. 2823. Sheep were pre-anestetized with Acethyl Promazine (Prequillan, Fatro, Ozzano dell’Emilia, Italy), 1 ml IM and anesthetized with sodium thiopental (10 mg/kg BW, Penthotal Sodium, Intervet srl, Milano, Italy). These treatments alleviate level of suffering to minimum. After surgery animals were kept in warm and dry place, isolated from animals until recovery.

### *In vitro* maturation (IVM)

Sheep ovaries were collected from local slaughterhouses and transferred to the laboratory within 1–2 hours. Oocytes were aspirated with 21 G needles in presence of TCM-199 medium (Gibco, Milan, Italy) containing Hepes and 0.005% (w/v) Heparin. Then, all oocytes with an unexpanded cumulus and uniform cytoplasm were *in vitro* maturated (IVM) in bicarbonate-buffered TCM-199 medium (Gibco Milan, Italy) containing 2 mM glutamine, 0.3 mM sodium pyruvate, 100 μM cysteamine, 10% fetal bovine serum (FBS) (Gibco Milan, Italy), 5 μg/ml FSH (Ovagen, Glenfield, New Zeland), 5 μg/ml LH, and 1 μg/ml estradiol. Maturation was conducted into 4-well culture plates (Nunclon, Roskilde, Denmark) containing 0.4 ml of IVM medium per well and incubated in a humidified atmosphere of 5% CO_2_ in air at 39°C for 24 h.

### *In vitro* embryo production (CTR)

*In vitro* fertilized (IVF) embryos were produced as previously described [23] and were used as a control group in all experiments (CTR). Briefly, matured oocytes were partially stripped of cumulus cells by repeated pipetting. Frozen semen was rapidly thawed at 37°C and washed twice by centrifugation at 500 g for 5 min with bicarbonate-buffered SOF with 4mg/ml BSA. IVF was carried out in 50 μl drops under oil, using 5 × 10^6^ cell/ml and a maximum of 15 oocytes per drop, at 38.5°C in 5% CO_2_ for 20 h. The IVF medium was bicarbonate-buffered, SOF enriched with 20% (v/v) heat-inactivated estrous sheep serum, 2.9mM Ca2+ lactate, and 16 μM isoproterenol. Presumptive zygotes were transferred into 20 μl drops of SOF enriched with 1% (v:v) Basal Medium Eagle (BME) essential amino acids, 1% (v:v) Minimum Essential Medium (MEM), non-essential amino acids (Gibco, Milan, Italy), 1mM glutamine and 8 mg/ml fatty acid-free BSA (SOFaa-BSA) under oil. Zygotes were cultured in a humidified atmosphere of 5% CO_2_, 7% O_2_, 88% N_2_ at 38.5°C, and the medium was changed on day 3 (supplemented with glucose) and day 5 (supplemented with 10% FBS charcoal stripped). Cleavage was assessed on day 1 and blastocyst formation was recorded on day 6 of culture.

### Somatic cell nuclear transfer (SCNT)

SCNT was performed as previously described [24]. Briefly, mature sheep oocytes were incubated in Hepes-buffered TCM-199 medium containing 4 mg/ml BSA, 7.5 mg/ml Cytochalasin B and 5 mg/ml Hoechst 33342 in an incubator for 15 minutes. Oocyte manipulation was facilitated by Piezo pulses (PiezoXpert, Eppendorf, Milan, Italy). Enucleation was carried out in Hepes-buffered TCM-199 medium with 0.4% (w/v) BSA and Cytochalasin B by using a Narishighe micromanipulator. Enucleated oocytes were allowed to recover from the Cytochalasin B treatment and then were directly injected with fibroblasts in PBS with 6% Polyvinylpyrrolidone. Reconstructed oocytes were activated in Hepes-buffered TCM-199 medium containing 5 mg/ml Ionomycin for 5 minutes and then incubated in SOF medium plus antibiotics and 0.8% BSA containing 10mM dimethylaminopurine and 7.5 mg/ml Cytochalasin B for 3–5 hours. Then embryos were cultured for 10–12 hours in SOF enriched with 1% (v:v) MEM nonessential amino acids (Gibco), 2% (v:v) basal medium Eagle (BME) essential amino acids, 1 mM glutamine, and 8 mg/ml BSA covered with mineral oil pre-washed in SOF.

### Animal treatment, embryo transfer, and sample recovery

#### Animal treatment and care

Sardinian ewes (n = 30) obtained from local breeders were housed in the authorized experimental farm from the Istituto Zooprofilattico Abruzzo, Loc. Gattia, Italy, feed and kept under the best sheep housing standards. The synchronization of sheep was achieved with Crono-gest sponges of 25 mg (Intervet, Milan, Italy). After 12 days Crono-gest sponges were removed and estrous monitored for 48h. Six days after estrous, embryo transfer was performed. Ewes were fasted for 24h before surgery and then were pre-anestetized with 1 ml IM Acethyl Promazine (Prequillan, Fatro, Ozzano dell’Emilia, Italy) and anesthetized with sodium thiopental (10 mg/kg BW, Penthotal Sodium, Intervet Srl, Milano, Italy). These treatments alleviate level of suffering to minimum. After surgery animals were kept in warm and dry place, isolated from animals until recovery. Post-operatory suffering alleviation was induced by flumixin meglumine (Zoetis, Rome, Italy), given 1M, and antibiotic treatment consisted of intramuscular injection of ampicillin (0.2 g/10 kg, Amplital Vet, Ceva SpA, Agrate Brianza, Italy) every 24 hours for 3 days.

#### Embryo transfer

Sheep were divided into two groups: the first were recipients of SCNT, and the second of i*n vitro* produced (CTR) embryos. CTR and SCNT blastocysts (2–4 per ewe) were surgically transferred to the recipient ewes (n = 10: CTR; n = 15: SCNT) 6 days after estrus by paramedian laparatomy. After the exposition of uterine horns, a smooth catheter was introduced into the lumen, and embryos were deposited. After surgery animals were recovered as describe above.

#### Sample recovery

Fetuses and placentae of both groups were recovered by para-median laparotomy at 20–22 days of gestation. Once collected in Petri dishes (90 mm) with warm Ca^2+^/Mg^2+^ PBS containing 0.005% (w:v) heparin, fetuses were observed under the stereomicroscope to assess their vitality. Only those with hearth beating were consider for subsequent analysis. There were recovered n = 8 CTR and n = 5 SCNT fetuses (80% and 33% pregnancy rate, respectively). Placental samples were snap frozen in liquid nitrogen and stored for subsequent analysis and/or fixed for histological and ultrastructural evaluation. At the end of experiments, animals were scarified according to the national regulation.

### Western blotting

Total mitochondrial proteins from placental tissues (n = 3 for both CTR and SCNT) were extracted using a Mitochondrial Isolation Kit for Tissue (Abcam, Cambridge, UK) according to the manufacturer’s instructions. Then proteins were denatured by heating at 95°C for 5 min in 1% (v:v) sodium dodecyl sulphate (SDS), 1% (v:v) β-mercaptoethanol, 20% (v:v) glycerol in 50 mM Tris–HCl at pH 6.8. Samples were subjected to electrophoresis in 10%SDS polyacrylamide gels. After electrophoresis, proteins were transferred to nitrocellulose membranes. Membranes were blocked in TBS-T (0.2% (v:v) Tween-20 in 20 mM Tris, 137 mM NaCl at pH 7.6) with 5% (w:v) skimmed milk for 1h at room temperature (RT). Then, membranes were incubated with the primary mouse monoclonal anti-Mfn2 (1:300; sc100560, Santa Cruz Biotechnology, Heidelberg, Germany), rabbit polyclonal anti-Bnip3L/NIX (1:300; Abcam ab8399, Cambridge, UK); and goat polyclonal anti-actin (1:1000; sc-1615, Santa Cruz Biotechnology, Heidelberg, Germany) antibodies diluted in 0.1% blocking solution at 4°C overnight (O/N). After three washes with TBS-T, membranes were incubated at RT with the secondary antibodies (1:1000) in 0.1% blocking solution for 1h. After three washes in TBS-T, the final detection was performed by enhanced chemiluminescence using the ECL Plus Western Blotting Detection System (Amersham, Milan, Italy). Image acquisition was carried out using the ChemiDoc System (Bio-Rad, Milan, Italy).

### Transmission electron microscopy (TEM)

Placental tissues from both experimental groups (n = 4 for both CTR and SCNT) were washed twice with PBS and fixed in glutaraldehyde (2.5% in 0.1 M cacodylate buffer, pH 7.4) for 24 h. After washing in ddH_2_O, cells were post-fixed in 2% OsO_4_ in ddH_2_O for 4 h and washed three times in ddH_2_O. Next, cells were dehydrated through a graded series of ethanol solutions (30%– 10 min, 50%– 15 min, 70%– 24 h, 80%– 10 min, 96%– 10 min, 100%– 10 min, acetone—twice for 15 min) and were infiltrated with graded concentrations of EPON resin in 100% acetone (1:3–20 min, 1:1–24 h, 3:1–2 h), infused twice for 1 h in pure EPON resin and polymerized at 65°C for 24 h. Next, 60 nm sections were prepared and examined using a LEO 912AB electron microscope. Images were captured using a Slow Scan CCD camera (Proscane, Lagerlechfeld, Germany) and EsiVision Pro 3.2 software (Soft Imaging Systems GmbH).

### Histological analysis

Chorion-allantois tissues (n = 4 for both CTR and SCNT) were fixed in 4% (w:v) paraformaldehyde and subsequently dehydrated into increasing ethanol solutions for 5 minutes in each step and then cleared in xylene mixture. Finally, placentae were paraplast embedded. For hematoxylin and eosin staining, 5 μm sections were used. Pictures were taken using the Nikon Eclipse E600 microscope (Nikon, Milan, Italy).

### Binuclear cell counting

The number of binuclear cells per field was calculated in SCNT and CTR histologically prepared early placentae. The number of binuclear cells was evaluated by randomly analysing 15–20 fields per sample under a microscope with immersion (100x). Binuclear cells were considered to be characteristic if they contained two randomly scattered nuclei or if they occurred in small clusters, located deep within the trophoblastic layer.

### Expression analysis

Total RNA from placental tissues (n = 5 for both CTR and SCNT) was extracted using an SV Total RNA Isolation System (Promega, Milan, Italy) according to the manufacturer’s instructions. Total RNA integrity was assessed by a 2100 Bioanalyzer (Agilent Technologies). Samples with an RNA Integrity Number of at least 8.5 were used for subsequent analysis. All samples were reverse-transcribed using GoScript^™^ Reverse Transcription System (Promega, Milan, Italy) according to the manufacturer’s protocol. The obtained cDNAs were used for gene expression analysis using specific 5’-3’ primer pairs designed to anneal at 56–58°C with an amplification efficiency (E) range between 2.1 and 1.9 (available on request). Real-time PCR was carried out using SsoAdvanced Universal SYBR Green Supermix (Bio-Rad, Milan, Italy) with a CFX Connect Real-time PCR detection system (Bio-Rad, Milan, Italy), according to the manufacturer’s instructions. Relative gene expression data were calculated using the comparative threshold cycle method (ΔΔCt) with GAPDH, μTUBULIN, and SDHA as housekeeping genes.

### Statistical analysis and software

All reported data are expressed as mean with relative standard error of mean (SEM). Decimal variables were analyzed using a Mann-Whitney test, while variables expressed as percentage were analyzed with Fisher’s exact test. Plots of the probability value (p) has been realized using GraphPad (PRISM software version 3.03). Immunofluorescence results were obtained using Image J software. Primer sets were designed using the Primer3 tool, reference stability values were calculated using GeNorm, and efficiency values and data analysis of the amplification runs were performed using BioRad software.

## Results

### Defects in somatic cell nuclear transfer (SCNT) placenta

**Fig 1:**
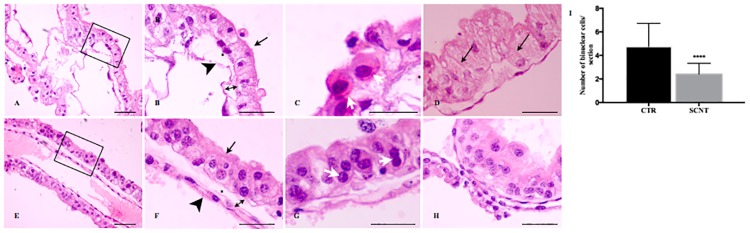
Abnormalities in sheep SCNT placentae. (A-D) Haematoxylin and eosin staining of placentae collected from SCNT and (E-H) control (CTR) embryos at an early stage of development; (A) SCNT placentae at magnification x20 and (B) in enlarged black square from picture A. Chorion present cuboidal and/or columnar cells with poor cell organisation (arrow) and high cytoplasmic vacuolisation. Cells had apically situated and very shrunk nuclei. Allantois was consisted by low cells number (B); thin and frayed basement membrane (star) as well as irregular connective tissue were observed (arrow double heads); (C, G) Binuclear cells (white arrows); (D) Cytoplasmic vacuolisation in placentae collected from SCNT embryos (arrows); (E) Haematoxylin and eosin staining of CTR placenta in magnification x20 and enlarged black square from picture E; (F) Well-developed chorion (black arrow), and allantois (black arrowhead) were observed. Chorion presents cuboidal cells, with poor cellular organisations and few empty, cellular vacuoles, situated on well visible basement membrane (black star). Distance between chorion and allantois was regular and shorter than SCNT once (double arrowheads); (H) Histological staining of CTR early placenta; (I) Graph presents number of binuclear cells in placentae collected from SCNT and CTR embryos at an early day of development; p<0.001. Scale bars represents: 100μm for (A) and (E); 50μm for (B, C, D, F, G, H).

Histological analysis of SCNT embryos revealed a disrupted cellular architecture compared to control embryos (Fig 1). In particular, SCNT placentae showed the columnar chorion cells on a frayed and thin basement membrane (Fig 1A and 1B, arrow). Nuclei were stacked in different positions with the chromatin shrunk in most of the cells, a finding typical of cellular death (Fig 1B). The connective tissue was likewise abnormal, with reduced vertical collagen filaments (Fig 1B, black star; between chorion and allantois), while collagen filaments were mostly horizontally oriented in CTR (Fig 1E and 1F, black star). The gap between chorion and allantois was significantly greater in SCNT extra-embryonic tissue than in CTR samples (Fig 1B vs 1F, double-headed arrow), a finding indicative of loss of functional coupling between these tissues. An in-depth analysis of the SCNT allantois revealed a prevalence of irregular and poorly organized layers of cells (Fig 1B, arrowhead). In contrast, CTR chorion displayed a mono- or bilayers of cuboidal-columnar cells with basally located nuclei (Fig 1D and 1E) placed on a well-developed basement membrane (Fig 1F, black star). The allantois of CTR placentae was well organized and composed of many thin, elongated cells (Fig 1F, arrowhead).

An in-depth phenotypic characterization of placentae was undertaken by focusing on the morphology of extra-embryonic sheep tissue and by counting the number of binuclear cells. Histological sections of SCNT placentae were dominated by immature binuclear cells located on the surface of the chorion (Fig 1C, white arrows), which appeared as small round cells with darker cytoplasm surrounding mononuclear trophoblast cells; mature cells were rarely observed. In contrast, CTR placentae presented mature, characteristic binuclear cells (Fig 1G), randomly scattered and located deep within the trophectodermal layer (Fig 1F and 1G). The number of nucleated cells was statistically lower in SCNT placentae compared to CTR ones (1 binuclear cell/ section vs. 3.5 binuclear cells/section, respectively, p<0.0001) (Fig 1I).

### Early SCNT placentae have aberrant mitochondrial and endoplasmic reticulum (ER) morphology

**Fig 2:**
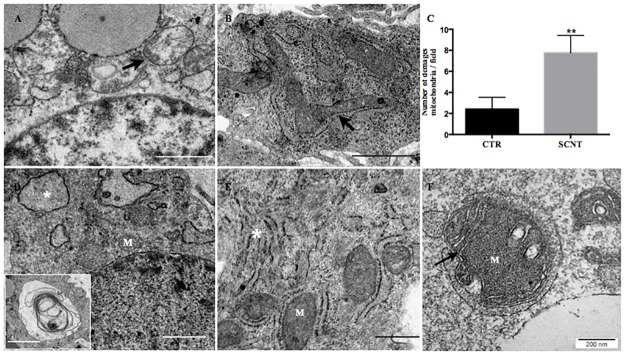
Ultrastructural defects in placentae collected from sheep SCNT embryos. (A) Damaged mitochondria in SCNT placentae; (B) Healthy mitochondria in CTR placenta, mitochondria in fusing process (arrow); (C) Number of damaged mitochondria per field in SCNT and CTR early placentae; (D) swollen ER (white stars) in SCNT early placenta; (E) Healthy ER in CTR placentae (F) Incomplete incorporation of damaged mitochondrion (M) into autophagosome (arrow) in SCNT placenta. Scale bars represent (A, B and D) 1000 nm; (D) 2000 nm; (E) 500 nm and (F) 200 nm.

Drastic differences in mitochondrial structure between early SCNT and CTR placentae were observed in the TEM analysis. Most mitochondria in SCNT placentae were randomly dispersed, swollen, fragmented, and devoid of cristae (Fig 2A, arrow), while mitochondria in CTR placental tissue displayed a canonical structure, with a round to ovoid shape and mature and evenly stacked cristae (Fig 2B). The normalcy of the mitochondria in CTR placentae was also underlined by the observation of many mitochondria engaged in the fusion process (Fig 2B, arrow), which never occurred in SCNT placentae. Moreover, damaged mitochondria accumulated in extra-embryonic tissue of SCNT conceptuses, without being included into the autophagosome, as normally occurs (Fig 2C and 2F). Beside mitochondrial abnormalities, TEM analysis also showed drastically swollen autophagosome membranes (Fig 2F, arrow) and dramatic expansion of the ER (Fig 2D, white star), characterized by many multilamellar structures in the cloned placentae (Fig 2D, white square).

### Mfn2 level is decreased in the early SCNT placenta

**Fig 3:**
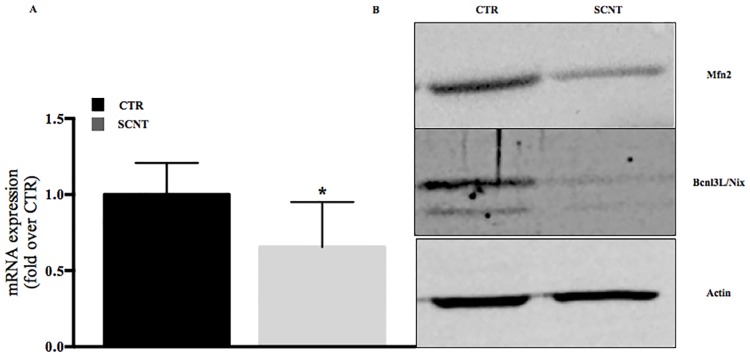
Mitochondrial protein expressed in sheep placentae. (A) mRNA expression of *Mfn*2 in SCNT and CTR early placentae, p = 0.025; (B) Protein expression of Mfn2 and Bcnl3L/Nix in early SCNT and CTR placentae. Actin was used as a loading control.

Ultrastructure results may suggest inappropriate functions of mitochondria. Moreover, mitochondrial fusion was rarely observed in the TEM analysis of SCNT placentae. Mitochondrial fusion is a critical mechanism that plays a crucial role in the proper function of those organelles. The protein primarily responsible for the fusion process is mitofusin 2 (Mfn2). To determine whether the lack of mitochondrial fusion and observed mitochondrial abnormalities are correlated with Mfn2 expression, mRNA and protein in early SCNT placenta were analysed. The data set showed statistically lower expressions of mRNA (Fig 3A) (p = 0.025) and protein (Fig 3B) in SCNT placentae. These findings suggest that deregulated expression of Mfn2 protein in SCNT placentae affect abnormal mitochondrial morphology and function.

### Low expression of Bcnl3L/Nix in SCNT placentae

**Fig 4:**
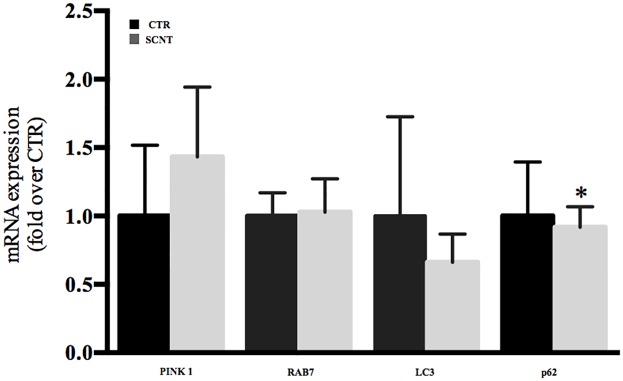
Expression of autophagic and mitophagic genes in sheep SCNT placentae. *p62* was statistically lower in the SCNT group (p>0.05), while other analysed genes were not different from CTR group.

We next examined whether damaged mitochondria may be efficiently removed from the cells, by investigating the expression of the mitochondrial outer membrane Bcnl3L/Nix protein. Nix protein plays a crucial role in the selective elimination of broken/damaged mitochondria from cells. The results showed very low expression levels of Nix protein in SCNT placentae (Fig 3B), which suggests a lack of clearance of damaged mitochondria. Fig 2F clearly shows a damaged, swollen, poorly-developed cristae mitochondrion, not efficiently incorporated into autophagosomes (Fig 2F, arrow). The next step was to evaluate genes playing crucial roles in autophagy and mitophagy process. Interestingly and surprisingly, from the analyzed autophagic/mitophagic genes (*LC-3*, *Rab7*, *p62* and *PINK 1*), only *p62* was statistically different in the SCNT group (p>0.05) (Fig 4).

### Low expression of mitochondrial proteins is associated with apoptosis in the SCNT placenta

**Fig 5:**
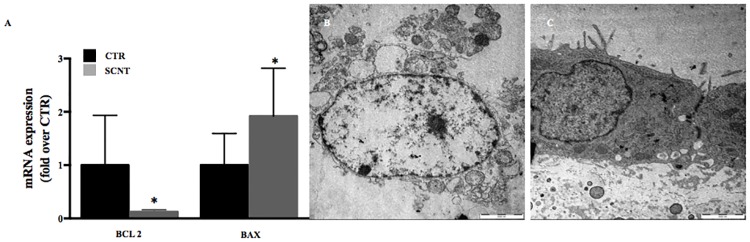
Apoptosis in sheep SCNT placentae. (A) mRNA expression of apoptotic genes (*BCL2* and *BAX*) in SCNT and CTR placentae; * p>0.05. (B) Apoptotic cell in SCNT placentae and (C) normal chorion cell in CTR group. Scale bars 2000 nm.

To assess whether the perturbed mitochondrial functions are associated with apoptosis in the SCNT placentae, we next analyzed the mRNA expression of the anti-apoptotic *BCL2* and pro-apoptotic *BAX*. The results revealed that levels of *BCL2* were significantly lower (p = 0.03) compared to the CTR group, while levels of *BAX* were significantly higher (p = 0.04) in SCNT placentae (Fig 5A). These gene expression data are in concordance with the ultrastructure phenotype of SCNT extra-embryonic tissue, with apoptotic features, fragmented cytoplasm, a high number of apoptotic bodies, condensed chromatin, as well as a lack of integrity of the nuclear membrane (Fig 5B).

## Discussion

Our previous work suggested that the abnormalities described for SCNT placentae might have features in common with mitochondrial dysfunction [9, 12]. Here, we report that deregulated expression of mitochondrial proteins (Mfn2 and Bcnl3L) cause placental abnormalities and may negatively affect SCNT embryo development. None of the mitochondrial functions addressed in this study was normal. Low expression of *Mfn2* mRNA and protein in early SCNT placentae, found here, caused aberrant mitochondrial morphology (Figs 2 and 3), as well as low expression of BNIP3L protein, which is responsible for selective elimination of damaged mitochondria (Fig 3B). These results overlap with those previously observed in human placentae collected from “unexplained” early miscarriages [21], as well as mouse dendritic cells mutant for Mfn2 [25], and mouse embryonic fibroblast-Mfn2 knockout cells [26].

SCNT fetuses usually die in early stages of development, just after implantation. Binuclear cells in sheep placentae—homologues to extravillous trophoblasts in humans and giant cells in rodents [27]–play crucial roles in implantation and the production of hormones, such as placental lactogen and progesterone [28, 29]. Here, we have found that impaired mitochondrial morphology may cause improper cellular function, due to poor development of binuclear cells (Fig 1C and 1I). In fact, Chan and colleagues have shown that mice deficient in Mfn2 die in mid-gestation, mostly as a result of specific and severe disruptions in the placental trophoblast binuclear cell layer [20]. Moreover, human extravillous trophoblastic cells from early miscarriages exhibit very low expression of mitochondrial fusion protein (Mfn2) and damaged mitochondria [21].

TEM analysis demonstrated that the contents of autophagosomes were frequently partially digested or undigested, suggesting that autophagosome maturation may be impaired in SCNT placentae (Fig 2F) [30]. An additional role of Mfn2 is facilitating contact between mitochondria and the endoplasmatic reticulum, finalized mainly by calcium exchange. Low levels of calcium in the cell may cause ER stress [31]. Our histological and TEM analyses demonstrated an extensive cytoplasmic vacuolization (Fig 1D) due to swollen ER (Fig 2C, white stars), which precludes proper autophagosome formation (Fig 2F). The controlled elimination of defective mitochondria is necessary for cell function and development [32]. Defects in this elimination process have been linked to aging, degenerative diseases, and cancer [33], and could also play a role in SCNT placental abnormalities. The mitochondrial outer membrane protein BNIP3L (also known as Nix) is required for the selective clearance and elimination of mitochondria [34, 35]. Low expression of Nix negatively affects incorporation of damaged mitochondria into autophagosomes [36,37], resulting in abnormal cellular function. In accordance with these previous findings, we have shown low expression of Nix proteins in early cloned placentae (Fig 3B). Those observations may suggest improper function of autophagy/mitophagy processes, one of which is the removal of damaged mitochondria from the cell. Thus, we next checked major autophagic and mitophagic genes. It has been shown that in Nix-deficient cells, mitochondria fail to enter autophagosomes, while autophagy per se is functional [34, 37]. We observed statistically lower expression of the *p62/SQSTM1* mRNA (Fig 4), which is involved in the last step of mitophagy, the formation of a complex with PINK-Parkin proteins [38]. Mitochondrial depolarization stabilizes PINK1 to recruit Parkin to damaged mitochondria and direct them for mitophagy. PINK1-Parkin can mediate ubiquitination of the mitochondrial surface Mfn2. Subsequently; p62/SQSTM1 may recognize multi-ubiquitinated Mfn2 on these mitochondria and recruit autophagosomes [39]. It has been suggested that Parkin-mediated poly-ubiquitination of Mfn2 induces mitophagy. Thus, the low expression of Mfn2 in SCNT placentae involves not only a deregulation of mitochondrial fusion, but also a deregulation of mitophagy, by failing to eliminate depolarized mitochondria [40] (Fig 4).

Surprisingly, all analysed autophagic genes (*LC-3*, *Rab-7*, and *PINK1*) were not different from the CTR group (Fig 4). In fact, knockout of autophagy-specific genes (*Ulk1*, *Atg5* and *Atg7*) in cells did not abolish fully programmed mitochondrial clearance (mitophagy) [41,42], suggesting the existence of autophagy-independent pathways for programmed mitochondrial clearance in cells.

Perturbed mitochondrial function and the ensuing cellular suffering (Fig 5) were likely consequences of lower expression of *BCL2* and higher expression of *BAX* in the SCNT group (Fig 5A). Mitochondrial fusion plays a protective role against apoptosis [43,44], and therefore Mfn2 loss-of-function increases sensitivity to apoptotic stimuli [32]. This increased sensitivity to apoptosis is associated with mitochondrial fragmentation [44].

It is commonly accepted that the low developmental potential of SCNT embryos results from incomplete nuclear reprogramming. The limited coding capacity of mtDNA necessitates a major contribution of protein from the nuclear genome [45]. It is estimated that 1,500–2,000 nuclear-encoded and cytoplasmatically translated proteins may be imported by mammalian mitochondria. Therefore, coordinated expression of the mitochondrial and nuclear genome is crucial in maintaining healthy and functional mitochondria.

Thus, our results suggest that the mitochondrial genome is incompletely reprogrammed and/or reactivated in SCNT embryos and this may cause placental abnormalities. Moreover, equally important observation is that mitochondrial dysfunction may cause placental abnormalities and has impact on SCNT pregnancy outcomes.
